# Molecular mechanism for thermal denaturation of thermophilic rhodopsin†
†Electronic supplementary information (ESI) available. See DOI: 10.1039/c9sc00855a


**DOI:** 10.1039/c9sc00855a

**Published:** 2019-06-20

**Authors:** Ramprasad Misra, Amiram Hirshfeld, Mordechai Sheves

**Affiliations:** a Department of Organic Chemistry , Weizmann Institute of Science , Rehovot 76100 , Israel . Email: mudi.sheves@weizmann.ac.il

## Abstract

Understanding the factors affecting the stability and function of proteins at the molecular level is of fundamental importance. In spite of their use in bioelectronics and optogenetics, factors influencing thermal stability of microbial rhodopsins, a class of photoreceptor protein ubiquitous in nature are not yet well-understood. Here we report on the molecular mechanism for thermal denaturation of microbial retinal proteins, including, a highly thermostable protein, thermophilic rhodopsin (TR). External stimuli-dependent thermal denaturation of TR, the proton pumping rhodopsin of *Thermus thermophilus* bacterium, and other microbial rhodopsins are spectroscopically studied to decipher the common factors guiding their thermal stability. The thermal denaturation process of the studied proteins is light-catalyzed and the apo-protein is thermally less stable than the corresponding retinal-covalently bound opsin. In addition, changes in structure of the retinal chromophore affect the thermal stability of TR. Our results indicate that the hydrolysis of the retinal protonated Schiff base (PSB) is the rate-determining step for denaturation of the TR as well as other retinal proteins. Unusually high thermal stability of TR multilayers, in which PSB hydrolysis is restricted due to lack of bulk water, strongly supports this proposal. Our results also show that the protonation state of the PSB counter-ion does not affect the thermal stability of the studied proteins. Thermal photo-bleaching of an artificial TR pigment derived from non-isomerizable *trans*-locked retinal suggests, rather counterintuitively, that the photoinduced retinal *trans*–*cis* isomerization is not a pre-requisite for light catalyzed thermal denaturation of TR. Protein conformation alteration triggered by light-induced retinal excited state formation is likely to facilitate the PSB hydrolysis.

## Abstract 2

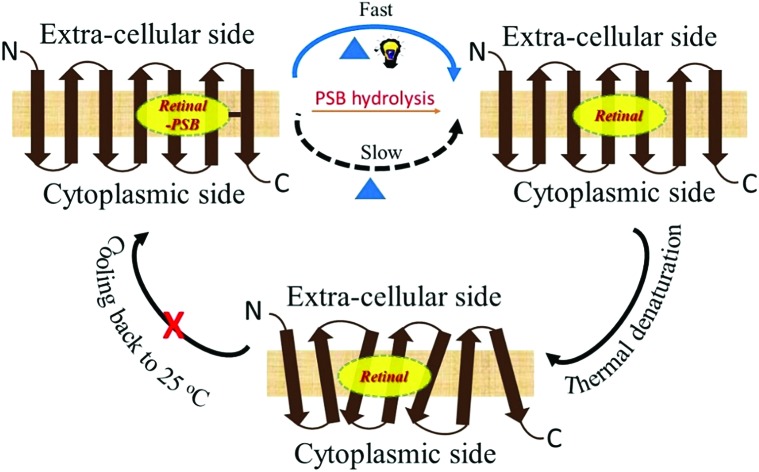
Studies of microbial rhodopsins revealed that hydrolysis of the retinal protonated Schiff base is the rate-determining step of the thermal denaturation process.

## Introduction

**Fig. 1:**
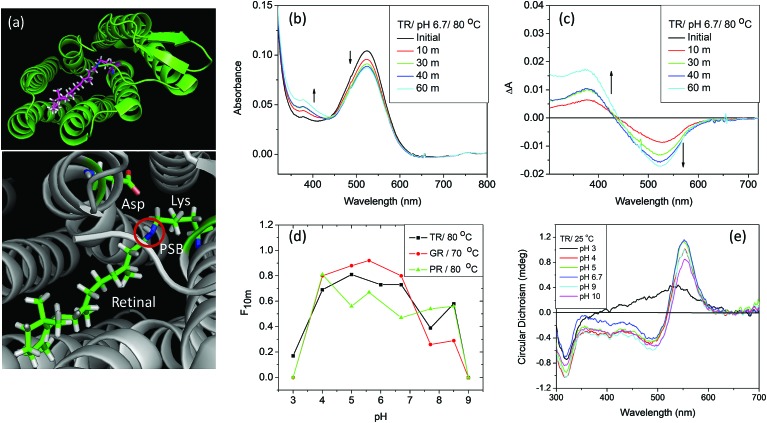
(a) General structure of microbial rhodopsins (upper panel), prepared from the crystal structure of TR (PDB ID: 5AZD). An all-*trans* retinal molecule is covalently linked to a lysine amino acid (Lys233 for TR) of the corresponding opsin through protonated Schiff base (PSB) formation. In TR, aspartic acid (Asp95) functions as the PSB counter-ion. (b) Change in absorption spectra of TR with time due to thermal denaturation of TR at 80 °C and pH 6.7 (50 mM phosphate buffer containing 0.06% DDM and 300 mM NaCl). (c) Difference spectra obtained by subtracting the initial spectrum of TR at 80 °C and pH 6.7. (d) The fraction of protein remained after 10 minutes of heating at 80 °C [F_10m_] for TR and PR and 70 °C for GR, compared to that in 25 °C. The curve shows that the protein is most stable in the pH range of 5–6. (e) The CD spectra of TR (25 °C) at pH 3, 4, 5, 6.7, 9 and 10. The CD spectrum of TR at pH 3 shows the characteristics of monomer, while that at pH 6.7 and 9 are trimers.

Rhodopsins, also known as retinal proteins, a class of photoreceptor proteins are ubiquitous in nature.^1^ The vertebrate rhodopsins (type-II), found in the eye's rod cell, are responsible for dim-light vision while the microbial rhodopsins (type-I) perform a wide range of functions, including, light-driven proton pump, ion channels and sensors.^2^–^5^ Rhodopsins are seven *trans*-membrane α-helical proteins containing a retinal chromophore, covalently bound to the ε amino group of a lysine residue (Lys233 for TR) of corresponding opsins through a protonated Schiff base (PSB) linkage (Fig. 1a). In spite of the striking resemblances of their secondary structures, and similarities in amino acid sequences, the thermal stability of microbial rhodopsins could be markedly different. For example, a recently discovered outward proton pump, thermophilic rhodopsin (TR) that shows 74% similarity in amino acid sequence to gloebacter rhodopsin (GR), exhibits much higher thermal stability than GR.^6^ TR, prepared from the genome of an extreme thermophile *Thermus thermophilus* bacterium living in a hot spring in the United States, showed high resistance to temperature change.^6^–^8^ The excited state photophysics of TR is reported to be independent of temperature up to about 70 °C.^9^ Microbial rhodopsins are being used in optogenetics, including, optical control of neural activity^10^,^11^ as well as in bioelectronic devices.^12^–^14^ Studies of thermal stability of animal as well as microbial rhodopsins have been reported since last few decades.^14^–^19^ The studies of thermal stability of retinal proteins gained momentum due to their potential technological applications in binary optical memories, as security ink and in solar cells.^12^–^14^ Proteins with desired thermal stability are prerequisites for many of such applications.

Retinal protein pigments undergo a photocycle upon photoexcitation that initiates their biological activity.^1^,^20^ The pigment photocycle consists of several photo-intermediates through which it reverts to the initial state. The pigment light absorption triggers isomerization of all-*trans* retinal to 13-*cis* retinal and 11-*cis* retinal to all-*trans* retinal in case of microbial and vertebrate rhodopsins, respectively. It was reported^19^ that the thermal denaturation of bacteriorhodopsin (BR) was accelerated in the presence of light. It was proposed that the denaturation of the protein under illumination occurs *via* a photo-intermediate, although any specific intermediate was not determined. The molecular mechanism for thermal stability of retinal proteins, and the exact factors that control the thermal stability are still not fully understood.

Here we have studied the factors affecting the thermal denaturation of TR, an outward proton pump, and other retinal proteins, *viz.*, GR, proteorhodopsin (PR) and BR. The denaturation process was followed by monitoring the absorption spectrum of the characteristic opsin covalently-bound retinal band. The thermal stability of the proteins studied is pH-dependent, and the thermal denaturation process is catalyzed by illumination. We propose that the hydrolysis of the PSB is the rate-determining step of the thermal denaturation process. Alteration of the retinal structure has profound effect on the thermal stability of TR. In addition, the light catalysis may be due to protein conformational alterations triggered by the retinal excited state.

## Materials and methods

### Preparation of the proteins and artificial pigments

TR,^9^ GR and PR are expressed as recombinant proteins in *Escherichia coli* and purified following previously reported procedure, described in the ESI.† TR apo-protein was prepared by reaction of freshly prepared hydroxyl amine with TR under illumination with white light using a cold light source (Schott, Germany) with <500 nm filter. The excess hydroxyl amine was removed in a centricon (4000 rpm, 20 °C), followed by washing it thrice using 0.02% *n*-dodecyl-β-D-maltoside (DDM) solution that also contained 300 mM NaCl. Reconstituted pigments were prepared by incubating either 1.5 equivalent of all-*trans* retinal or 1.5–2 equivalent of synthetic retinal to the apo-protein. Reconstitution of the retinals to TR apo-proteins is completed in about an hour, except for the *trans*-locked retinal which takes about a week to be completed. The modified retinals were synthesized using standard methods reported earlier.^21^,^22^ TR film was prepared by removing the solvent in a centricon (5000 rpm, 20 °C) and drop casting the highly concentrated solution inside a quartz cuvette of 10 mm path length. The solution was then dried in air, followed by water removal *via* vacuum pumping (Trivac, Model D4A; min. pressure: 1 × 10^–4^ Torr) and refilling the cuvette with argon.

### Sample preparation

We have used citrate (pH 3–4), phosphate (pH 5–8), Tris–HCl (pH 8.5) and carbonate (pH ≥ 9) buffer solutions of 50 mM concentration to maintain the desired pH values of the samples. Except for studies with BR, all the buffer solutions contain 0.06% DDM and 300 mM NaCl. 20% glycerol was added to the samples in order to minimize the effect of scattering during heating in the absorption spectra.

### Spectroscopic studies

The UV-visible absorption spectra of the samples were recorded in Agilent 4583 diode-array spectrophotometer (Agilent Technologies, Palo Alto, CA), equipped with an Agilent 89090A thermostated cuvette holder as temperature controller. The CD spectra were recorded in a Chirascan CD spectrometer (Applied Photophysics). The recorded absorption and CD spectra were corrected for scattering and baseline, respectively. Irradiation of samples were done using a Schott 250W cold light source equipped with a heat-absorbing filter and a level 4B optical fiber. Differential scanning fluorimetry (DSF) experiment was done using a nano-DSF instrument (Prometheus NT.48). The ratio of emission intensity at 330 nm to that of 350 nm of tryptophan (Trp) residue was recorded at 1 °C per minute rate. The melting temperature (*T*_m_) is the mid-point of the transition. The loss optical density of corresponding retinal covalently-bound opsin due to heating was fitted to the following single-exponential fitting function (1).

1 *y* = *a*e^–*kx*^

where, *a* represents the fraction of a component with rate constant *k*. All the fittings were done in Origin Lab 7.

### Titration experiment

The p*K*_a_ values were calculated using Henderson–Hasselbach eqn (2).^23^ Titration experiments were conducted using either HCl or NaOH of suitable concentration to achieve the desired pH. First, we raised the pH of the sample to about 11 and then gradually decreased it to about 1.5 and recorded absorption spectra at those pHs.
2

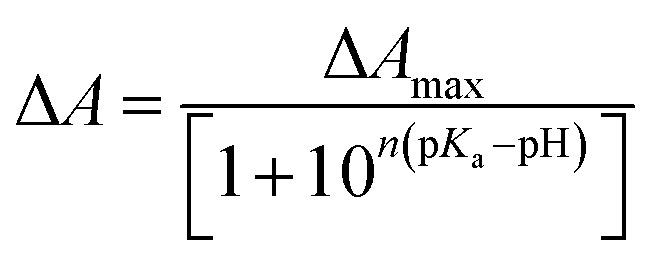

where, Δ*A* and Δ*A*_max_ denotes the absorbance difference and the maximum absorbance difference between protonated and deprotonated states, respectively. The term *n* stands for the number of protons participating in the transition and p*K*_a_ is the midpoint of the observed transition.

## Results and discussion

### External stimuli-dependent thermal denaturation of TR

TR, a detergent-soluble microbial retinal protein, was reported to be more thermally stable than many of its congeners.^7^,^8^ To understand which factors influence the thermal stability of this protein, we have studied the effect of pH alteration on its thermal denaturation. Alteration of the medium pH can change the protonation/deprotonation state of the PSB counter-ion, thereby altering the strength of hydrogen bond between PSB and its primary counter-ion (Fig. 1a). It has been recently proposed that nature of this hydrogen bond controls the ratio of reactive and non-reactive excited states in the sodium ion pump rhodopsin KR2.^24^ The proteorhodopsin (PR) was reported to function as a proton pump only when the counter-ion of PSB is in the anionic form.^25^ The effect of pH on several properties of vertebrate and microbial rhodopsins, including the thermal stability were previously reported.^16^,^26^–^29^ However, the effect of protonation/deprotonation state of the primary counter-ion of microbial rhodopsins on the thermal stability is not clear. Therefore, we have checked the effect of protonation of PSB counter-ion on the thermal stability of the studied proteins. We have studied the thermal stability of TR at different pHs in the temperature range of 70–90 °C. At pH 7, the protein is highly stable at 70 °C while at 90 °C it degraded comparatively faster as evident from loss of characteristic opsin-bound retinal absorption band at 530 nm (Fig. S1†). We have compared the thermal stability of TR at different pHs at 80 °C. The gradual change in absorption intensity of TR at 80 °C at pH 6.7 is shown in Fig. 1b, while that of pH 3 and 7.7 are shown in Fig. S2.† The difference spectra at pH 6.7, obtained by subtracting the initial spectrum of TR at 80 °C to those at different times (Fig. 1c), reveal a decrease in 530 nm band with a concomitant increase in the 377 nm band. The difference spectra indicate similar phenomenon for all other pHs (Fig. S2†), although the absorption maxima of the pigment band (*λ*_max_) vary due to pH change. The rate of loss of retinal absorption band at 530 nm shows exponential decay behavior. We found that the protein is most stable in the pH range of 5–6 and relatively less stable in the higher and lower pH regions. At pH > 9, most of the protein even degraded completely before reaching 80 °C. The fractions of TR that did not degrade following 10 minutes of heating at 80 °C at different pHs are shown in the Fig. 1d. The bell shape of pH *versus* thermal stability curve reveals that there are at least two protein residues, whose protonation states affect the thermal stability of the protein.

Circular dichroism (CD) spectroscopy is a versatile tool to study the structure of proteins.^30^ To monitor protein structural alterations following temperature change we have recorded the CD spectra of TR at different temperatures with varying pHs. The CD spectral studies show that TR at pH 6.7 has three bands in the visible region, of which one is positive with band at about 552 nm while the negative bands are at about 485 nm and 317 nm (Fig. 1e). It was reported^8^ that TR adopts a trimer form at room temperature and neutral pH, and irreversibly converts to monomeric form while heated above 68 °C. The protonation of the primary counter-ion as well as inter- and intramolecular interactions were proposed to affect the trimer-monomer equilibrium. We have shown^31^ earlier that the CD spectrum of TR(trimer) lost its characteristic bands irreversibly once heated to 80 °C due to formation of monomers that most likely eliminates the excitonic interaction among retinal chromophores in the trimer subunits. Fig. 1e shows that at high pH the protein maintains its trimeric structure. The protein does not show the characteristic bisignate CD spectrum of the trimer at pH 3, indicating that the protein adopts the monomeric form even at room temperature. At pH 4, TR forms a mixture of mostly trimeric and monomeric forms. The present CD spectral studies (Fig. S3†) show that TR(trimer) is transformed into the monomeric form before reaching 80 °C in the studied pH range. Our DSF studied (see below) also show that TR trimer is converted to monomer before denaturation. Therefore, at 80 °C one follows the denaturation rate of the TR(monomer) form and the present studies cannot evaluate the thermal stability of the trimeric form of TR.

**Fig. 2:**
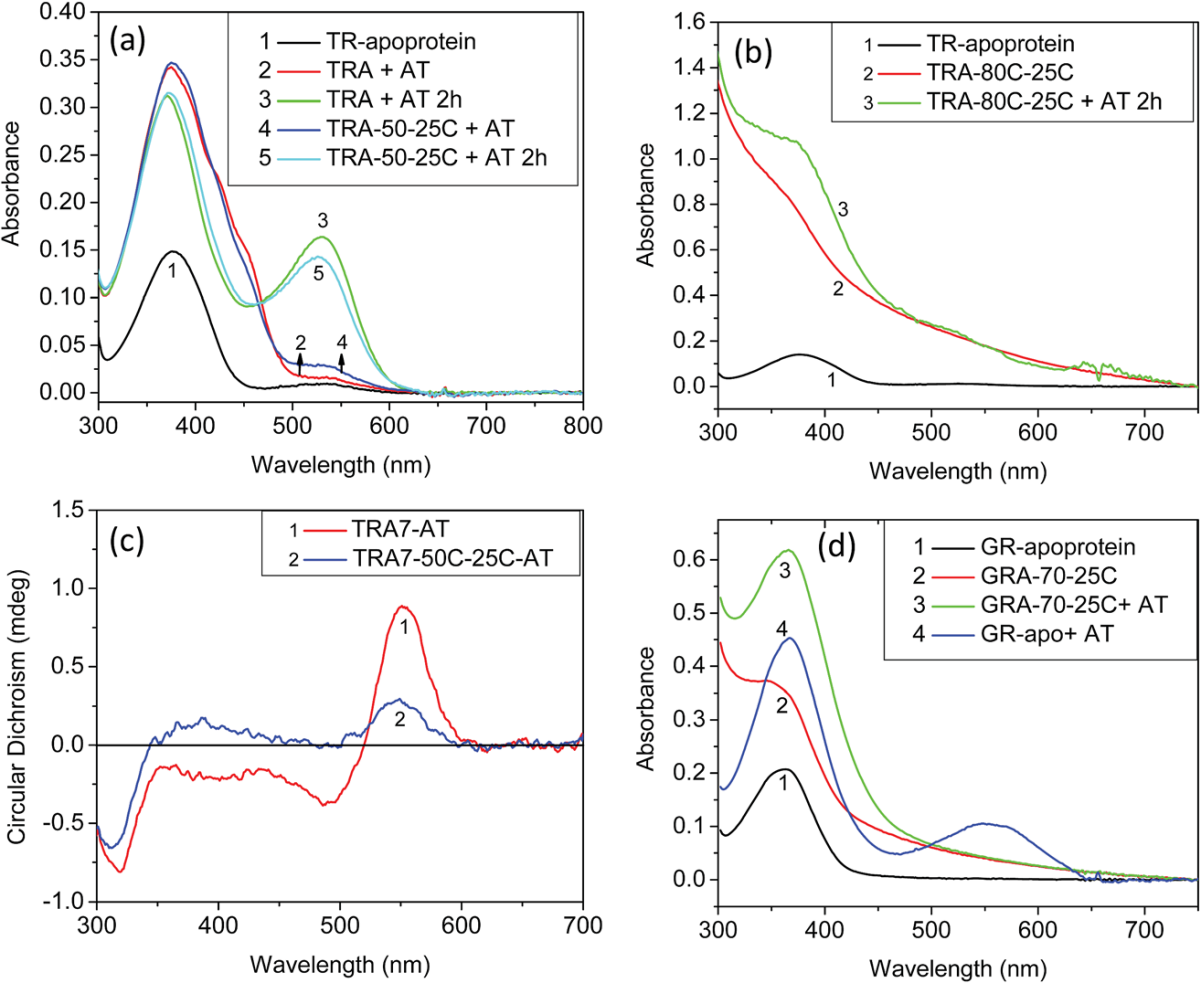
Absorption spectra of (a) TR apo-protein (**1**), immediately (**2**) and after 2 hours of addition of 1.5 equivalent of all-*trans* retinal (**3**). The absorption spectra of TR apo-protein cooled to 25 °C after heating to 50 °C, followed by addition of 1.5 equivalent of all-*trans* retinal (**4**) and after 2 hours of reconstitution (**5**). (b) TR apo-protein (**1**) heated to 80 °C, followed by cooling to 25 °C (**2**) and addition of 1.5 equivalent of all-*trans* retinal (**3**). (c) CD spectra of the reconstituted samples **3** and **5** shown in panel (a). (d) The absorption spectra of (**1**) GR apo-protein at 25 °C and (**2**) GR apo-protein heated to 70 °C, followed by cooling to 25 °C. The heated GR apo-protein does not bind to all-*trans* retinal (**3**), while the unperturbed GR apo-protein binds to all-*trans* retinal with good yield (**4**). All the experiment in this figure are done in pH 6.7 (50 mM phosphate buffer containing 0.06% DDM and 300 mM NaCl).

To understand the role played by the PSB linkage of the retinal to the protein stability, we have studied the thermal stability of TR apo-protein. The apo-protein at pH 6.7 was heated at 80 °C for 10 minutes, followed by cooling down to room temperature. The resulted apo-protein does not bind all-*trans* retinal even at room temperature while unperturbed apo-protein binds the retinal with very high yield (Fig. 2). This result indicates that the TR apo-protein is much less stable than the protein pigment in which the retinal is bound covalently. We further checked the melting temperatures (*T*_m_) values for thermal denaturation of TR and its apo-protein using differential scanning fluorometry (DSF) studies. The DSF studies show two *T*_m_ for TR at 71 and 86 °C, respectively at pH 6.7. We assign the transition at lower temperature to trimer to monomer transition while that at higher temperature is assigned to thermal denaturation of the protein. The TR apo-protein shows *T*_m_ values at 46 and 65 °C at pH 6.7 (Table S1†). To study whether the *T*_m_ at 46 °C of TR apo-protein represents the trimer to monomer transition, we incubated the TR apo-protein at 50 °C for 10 minutes, followed by cooling to 25 °C and binding all-*trans* retinal. The obtained protein is in its monomeric form as concluded from the CD spectrum (Fig. 2), indicating that the *T*_m_ of TR apo-protein at 46 °C indeed represents the trimer-monomer transition. Therefore, the DSF studies support as well the observation that TR apo-protein is less stable than the retinal covalently-bound protein. The DSF experiment shows only one transition with *T*_m_ at 81 °C for TR at pH 3, while two transitions are observed for other pHs (Table S1†). It is conceivable that TR at pH 3 is in the monomer form, and therefore the trimer to monomer transition is absent. In addition, we have performed CD measurements in the UV (200–240 nm) range that indicated as well that the TR apo-protein is less stable than native TR pigment (Fig. S4†). Experimental studies of folding of membrane proteins, including several retinal proteins, are limited especially in conditions in which protein denaturation is initiated.^32^–^34^ Several factors can affect the studies of thermal stability of membrane proteins including higher hydrophobicity of the transmembrane region, thermodynamics instability of the polar groups in the membrane and difficulty in refolding of the denatured protein. Recently, advent of several novel experimental tools, including, single-molecule dynamic force microscopy and atomic force microscopy assay facilitated studies of folding of membrane proteins, including, BR, the most studied microbial rhodopsin.^35^,^36^ It is known that CD spectra of proteins at the far UV region (UV-CD) of the electromagnetic spectrum gives rise to important information about their secondary structures, and can serve as a tool to detect denaturation mechanism.^37^,^38^ A protein with typical α-helical structure shows negative bands at 222 and 208 nm with a positive band at 193 nm, while a protein with well-defined β-sheet structure shows negative and positive bands at 218 and 195 nm, respectively (for details see a review by Greenfield^30^). The disordered proteins show low ellipticity above 210 nm with positive bands around 195 nm. Fig. S4† indicates that at 25 °C, TR shows characteristics of α-helical proteins. The secondary structure of this protein remains almost unchanged up to about 55 °C, whereas above it the percentage of β-sheet structure is gradually increased. At 90 °C, the protein has mostly β-sheet structure with signature of formation of disordered protein structure. A decrease in α-helical content of BR due to sodium dodecyl sulfate (SDS)-induced denaturation was reported earlier.^39^ For a quantitative analysis we have used web-based K2D3 software^40^ that analyses the relative contribution of the protein secondary (α-helix and β-sheet) structures derived from the measured UV-CD spectra (for a detailed procedure and error analysis, see Perez-Iratxeta *et al.*^41^). The K2D3 analysis shows (Table S2†) that UV-CD spectrum of TR at 25 °C is mostly composed of α-helix (75.8%) with a very small portion of β-sheet (1.4%). The contribution of the α-helix remains unchanged up to 70 °C, while a slight decrease is observed at 80 °C. At 90 °C, the contribution of α-helix structure to the observed CD spectrum decreases significantly with a consequential increase in the contribution of β-sheet structure. Similar analyses were performed on UV-CD spectra of TR apo-protein that showed that the apo-protein follows the same denaturation pathway, although it is less thermally stable than the corresponding retinal-covalently bound to the protein. The lower thermal stability of TR apo-protein detected by the CD spectroscopy echoes that observed by absorption and DSF experiments.

**Fig. 3:**
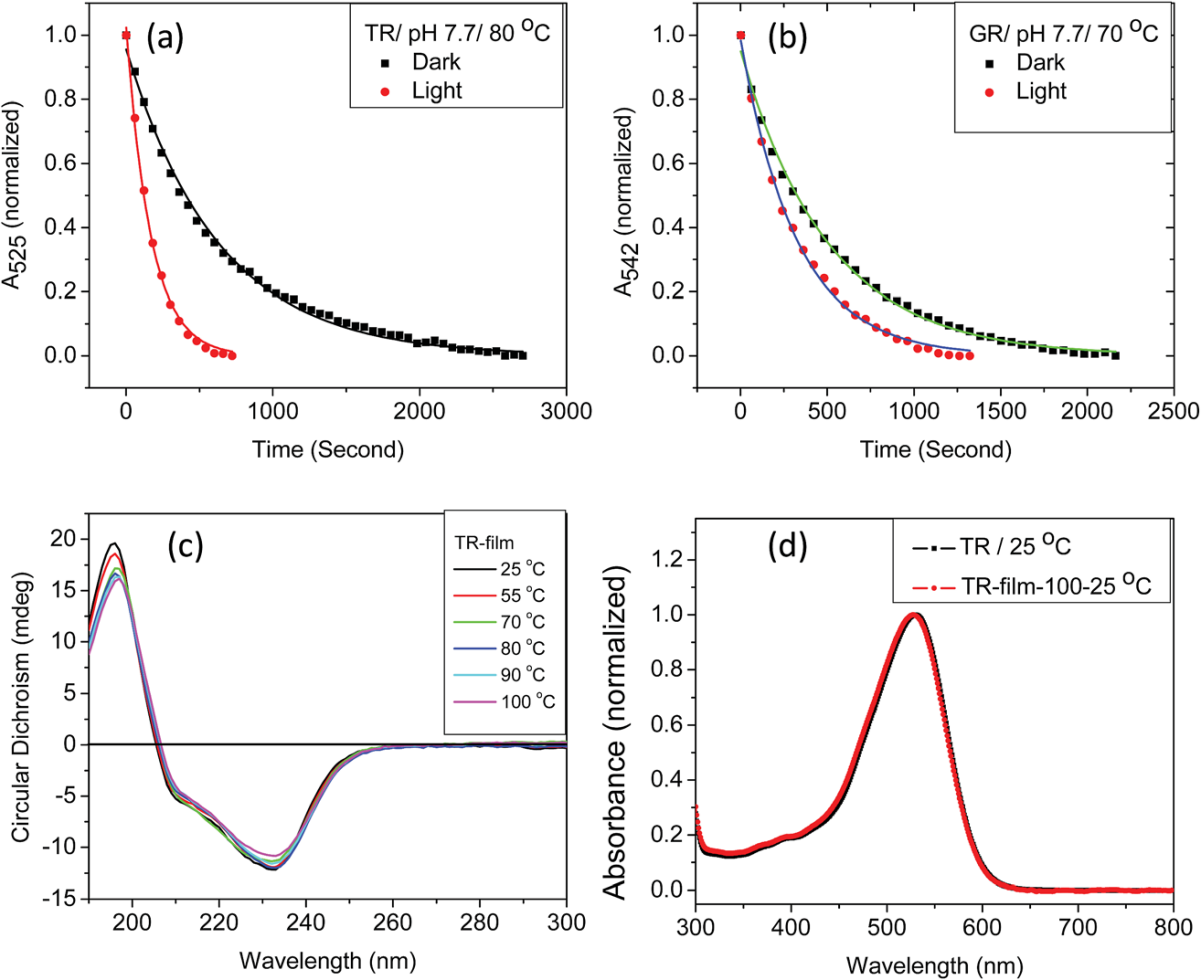
The effect of light on thermal denaturation of (a) TR and (b) GR at 80 and 70 °C, respectively at pH 7.7 (50 mM phosphate buffer containing 0.06% DDM and 300 mM NaCl). (c) The CD spectra of TR film at 25, 55, 70, 80, 90 and 100 °C (5 minutes at each temperature). (d) Comparison of normalized absorption spectra of TR film heated to 100 °C, followed by cooling to 25 °C and dissolved in a buffer of pH 6.7 (red) to its unperturbed counterpart (black). Absence of retinal band at 370 nm indicates no denaturation of TR multilayer at 100 °C.

We have found that the thermal denaturation of TR is accelerated using irradiation with white light. The effect of light on the denaturation of the protein at pH 7.7 and 80 °C is shown in Fig. 3, while those with varying pH and temperatures are shown in Fig. S5 and 6.† The effect of light on the protein denaturation process is more pronounced as the temperature is increased. The rate of the denaturation process of native TR is fitted to a single exponential decay eqn (1). The rate constants (Table 1) reveal that light accelerates the thermal denaturation by about 3 to 5 times, depending on pH and temperature. It is well-known^42^ that the reaction of the protein with hydroxylamine which forms retinal oxime and apo-protein can be accelerated by irradiation. The similarity between the hydroxylamine reaction and thermal denaturation of TR may suggest that these processes has some similitude in the corresponding reaction mechanism. We note that the temperatures of hydroxylamine reaction (25 °C) and thermal denaturation (80 °C) are widely different. Therefore, in spite of possible similarity in their mechanism the factors affecting these processes are different as the strength of physicochemical interactions among the protein subunits will be different at 25 and 80 °C. The three major observations described above can assist to pinpoint several factors that affect the thermal denaturation of TR. Firstly, the thermal denaturation process of TR can be accelerated using light irradiation. An analogy with hydroxyl amine reaction, which also can be catalyzed by illumination, supports the important involvement of the PSB hydrolysis in the denaturation process. Secondly, the difference spectra of thermal denaturation indicate formation of free retinal chromophore with concomitant decrease in TR pigment absorption band, indicating the hydrolysis of the retinal PSB to be a crucial step during thermal denaturation of the protein. Thirdly, absorption, CD and DSF studies show that the apo-protein is less thermally stable than the corresponding retinal-bound opsin. The latter observation indicates that the protein scaffold thermal stability is increased once the retinal is covalently bound to the protein. Therefore, the above described results strongly support that the hydrolysis of the retinal is the rate-determining step of the TR denaturation process.

**Table 1:** The rate constant obtained by fitting the degradation curves of TR and GR at pH 7.7 and at 80, 70 °C, respectively

Protein	*T* (°C)	*k* (s^–1^) × 10^–3^	*a*	*χ* ^2^
TR/Dark	80	1.6	0.96	0.996
TR/Light	80	6.0	1	0.997
GR/Dark	70	2.0	0.95	0.997
GR/Light	70	3.1	0.99	0.007

**Fig. 4:**
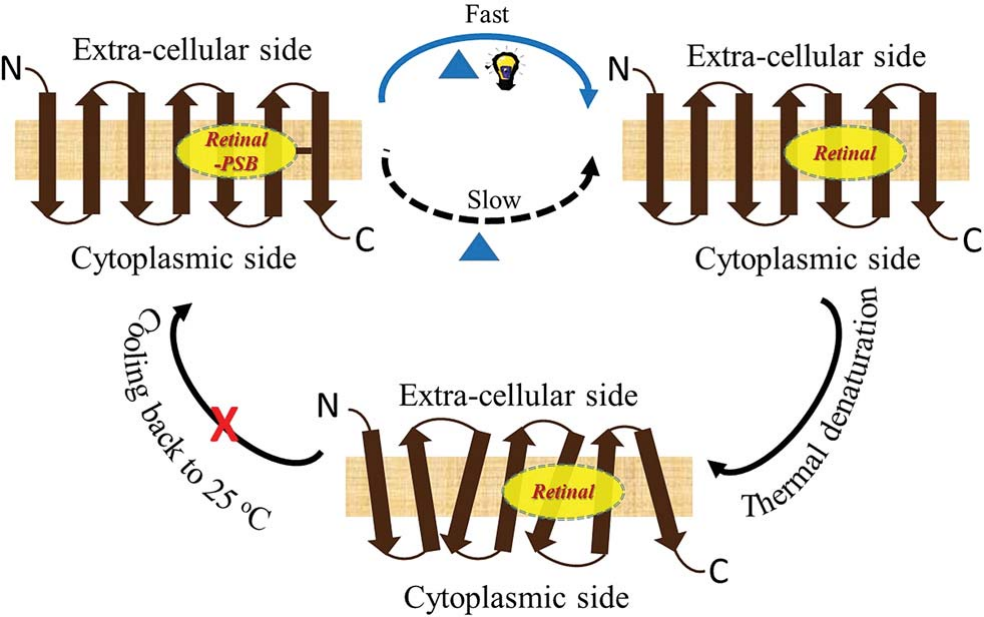
Schematic representation of thermal denaturation of microbial retinal proteins. In these proteins, an all-*trans* chromophore is covalently bound to a lysine residue through a protonated Schiff base (PSB) formation. The first step of thermal denaturation involves cleaving of PSB, forming apo-protein and retinal. This step can be accelerated using white light. The resulted apo-protein is thermally less stable than the protein with the covalently-bound retinal. The thermal denaturation of microbial retinal proteins is an irreversible process.

Studies by Sudo and co-workers suggested that the large number of hydrophobic residues is one of the factors responsible for greater thermal stability of TR.^43^ It is conceivable that the increase in hydrophobic residues in the vicinity of PSB restricts its hydrolysis, thereby exerting further stability to the protein. One may envisage that if the hydrolysis is restricted by removal of bulk water, the protein will exert higher thermal stability. Although the denaturation mechanism of a protein in the solid state could be different from that in solution, and can be affected by various factors, higher thermal stability of TR in multilayer film may provide a support to the necessity of PSB hydrolysis during thermal denaturation. Therefore, we have monitored by CD spectroscopy the thermal stability of TR in dry film, in which the hydrolysis is restricted. Our results (Fig. 3c) show that the TR in film did not experience any denaturation up to 100 °C. The heated TR film was then cooled down to 25 °C and resuspended in a buffer of pH 6.7. The absorption spectrum (Fig. 3d) did not show signature of denaturation of TR, confirming the result obtained in the CD measurements. In contrast to what observed in TR multilayer, the protein in solution of pH 6.7 denatured when heated to 90 °C (Fig. S1†). The higher stability of TR in film in which hydrolysis is restricted due to removal of bulk water strongly supports that hydrolysis is crucial for thermal denaturation of the protein. The proposed scheme of TR denaturation process is shown in Fig. 4.

### Thermal stability of other retinal proteins proton pumps

To study whether the above-described thermal behavior is unique for TR, we have studied the thermal denaturation of GR, PR and BR. Both GR (5.9) and PR (7.4) have higher pKa values of their PSB counter-ion relative to that of TR and BR. Therefore, if the protonation state of the PSB counter-ion plays a significant role in the thermal stability, the thermal stability of both GR and PR as function of pH is expected to be significantly different from that of TR. However, the pH *vs.* thermal stability curves (Fig. 1d) of GR and PR show similar behavior to that of TR. Therefore, it can be concluded that the protonation state of the PSB counter-ion does not significantly affect the thermal stability of the studied retinal proteins. The effect of pH on thermal stability of BR was studied previously.^29^ It was shown that change in pH has a minor effect on the pre-melting transition at 78–80 °C while the irreversible melting temperature was strongly affected once the pH was lowered below 6.5. It was proposed that the protonation state of carboxylic amino acids within the protein alters the hydrophobicity of certain regions of the protein. The protonation state also changes the strength of polar and hydrophobic tertiary bonds in both intra-helical and inter-helical regions. Therefore, the melting temperature of BR decreases with decreasing pH below 6.5. The change in pH also affects the cation binding capability of BR that in turn influences the thermal stability of the protein.^16^,^27^ It was proposed that the protein loses a color-controling cation at low pHs, thereby induces protein conformation alteration. This alteration decreases the denaturation temperature of BR to 65 °C. Our results suggest that the protonation state of the PSB counter-ion does not affect the thermal stability of TR, GR and PR proteins. Protonation of carboxyl groups at low pH or deprotonation of other unknown residues at high pH may decrease protein regions hydrophobicity that in turn facilitates efficient PSB hydrolysis. The question arises why the protonation state of the PSB counter-ion does not affect the protein thermal stability. It can be suggested that penetration of more bulk water to the protein interior and PSB vicinity due to protein conformation changes following increase in temperature affects PSB hydrolysis. Alternatively, raising the temperature may allow a nucleophilic attack of the strongly bound water in the PSB vicinity on the PSB. It is possible that these two plausible mechanisms of PSB hydrolysis are not affected by the counter-ion protonation state.

Similar to TR, that showed light catalysis of the denaturation reaction, the denaturation process of GR, PR and BR were accelerated by illumination (Fig. 3 and S5–6†). In addition, the thermal stability of the apo-protein of GR (GR-apo) was found to be less than its retinal-covalently bound analogue. The GR apo-protein was incubated for five minutes at 70 °C, followed by cooling to 25 °C. The resultant apo-protein did not bind all-*trans* retinal while reconstitution of all-*trans* retinal with high yield was achieved with the unperturbed apo-protein (Fig. 2). CD measurements of GR and its apo-protein at different temperatures revealed that GR is more stable than its apo-protein form (Fig. S4†). Analyses of the experimental UV-CD spectra of GR at pH 6.7 at different temperatures revealed that the protein shows mostly α-helical structure at 25 °C. The contribution of β-sheet structure is increased at high temperature (80 °C) with concomitant decrease in α-helical structure. Like TR, analyses of UV-CD results show that the apo-protein of GR is less stable than the corresponding retinal-bound protein. It was reported^44^ that BR retained its native folded structure even at 140 °C once incorporated in multilayer structures of self-assembled ordered films. We propose that the stability exerted by BR in films in vacuum is due to absence of water environment thereby hydrolysis in the films is prevented. Consequently, the thermal stability reflects the stability of the protein structure itself. These results further support our proposal that the hydrolysis of the PSB is the rate-determining step in the denaturation process of microbial rhodopsins. Therefore, it is possible to tune the thermal stability of microbial rhodopsins by restricting the PSB hydrolysis.

### Thermal stability of artificial TR pigments

**Fig. 5:**
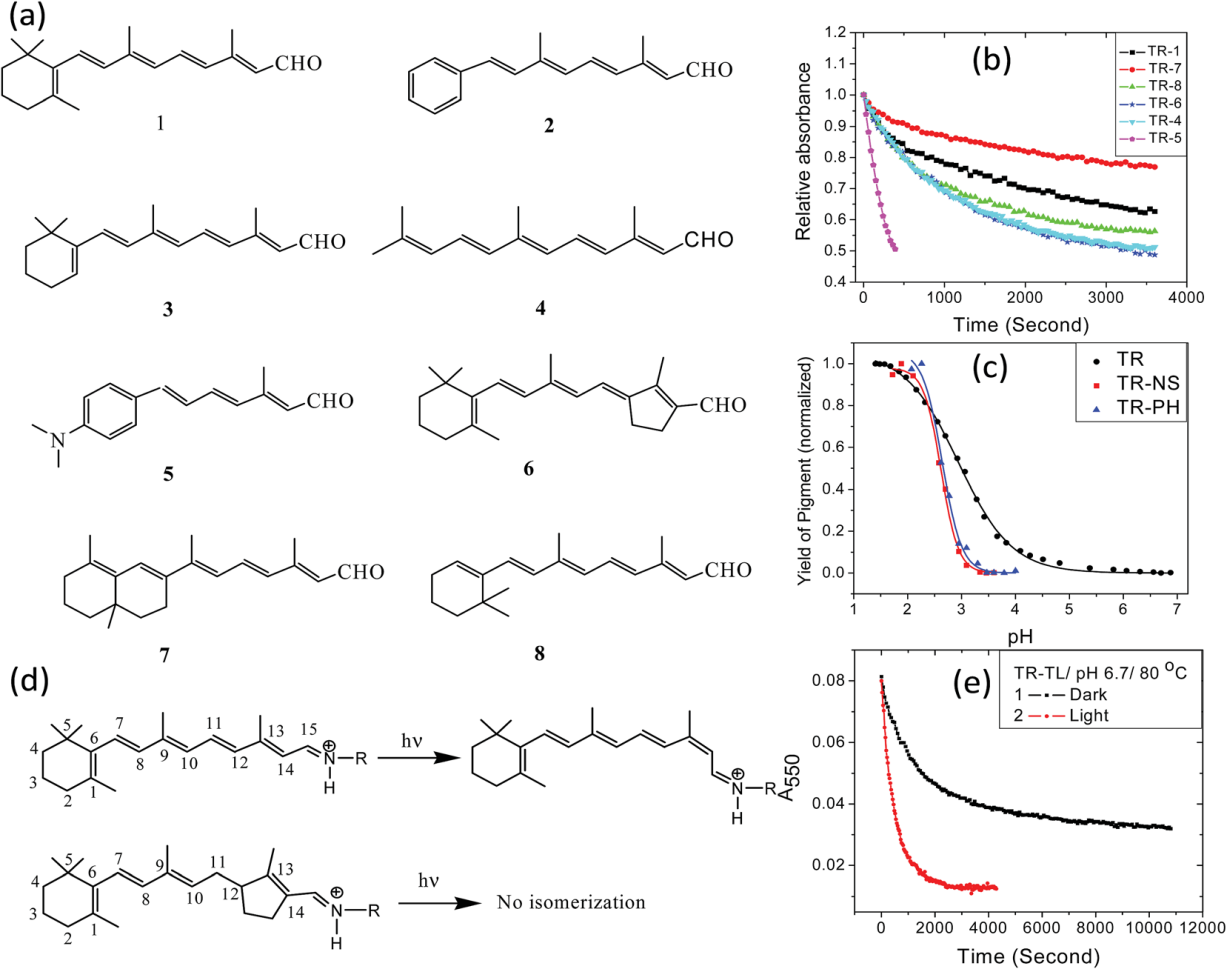
(a) Structure of all-*trans* retinal (**1**) and synthetic retinal analogs (**2–6**) used in the present study. The molecule **6** is *trans*-locked retinal in which the photo-induced C_13_=C_14_*trans*–*cis* double bond isomerization is restricted. (b) Decrease in pigment band of artificial TR pigments with time at pH 6.7 and 80 °C. (c) The change in absorption at 577, 636 and 545 nm for TR(trimer), TR-PH (**2** in panel a) and TR-NS (**5** in panel a) pigments due to change in pH, respectively. The solid lines represent the corresponding fitted curve. (d) The photo-induced isomerization of all-*trans* retinal, bound to the protein through a protonated Schiff base (PSB). Such isomerization is prevented in pigments derived from *trans*-locked retinal **6**. (e) The effect of light on the thermal denaturation of non-isomerizable *trans*-locked TR pigment at pH 6.7 and 80 °C. The change in absorption at 550 nm (*A*_550_) is plotted against time.

The retinal β-ionone ring resides in a protein hydrophobic environment. Modification of the ring electrostatic environment by introducing polar groups or poorly packed protein residues was reported to be detrimental for rhodopsin stability.^10^ To elucidate the effect of the retinal structure on the high thermal stability of TR, we have studied the thermal denaturation of artificial TR pigments derived from synthetic retinal analogs (Fig. 5a). The comparison of thermal stability of these artificial TR pigments show that replacing the β-ionone ring with phenyl or phenyl-amino group significantly reduced the thermal stability (Fig. 5b). Artificial TR pigments with phenyl ring (**2**, Fig. 5a) as well as that derived from aromatic retinal **5**, were denatured while heated to 80 °C. The artificial TR pigments derived from the bicyclic retinal **7** is the most stable among the studied artificial TR pigments. The retinal chromophore of several microbial retinal proteins was shown to adopt a ring-chain 6-*s-trans* conformation.^45^–^47^ The higher stability of the 6,7-locked retinal TR (**7**, Fig. 5a) suggests similar 6-*s-trans* conformation of the retinal in this protein too. To study whether the reduced stability was caused by alteration of the PSB counter-ion p*K*_a_, we have spectroscopically titrated the artificial TR pigments with synthetic retinals **2** and **5** (Fig. 5c). The p*K*_a_ of the counter-ion in the artificial TR pigments remained almost unchanged relative to wild-type TR (2.6 for TR2, compared to 2.9 for wild-type TR). This led us to infer that the pKa of primary PSB counter-ion is not the major factor responsible for the decreased stability of the artificial TR pigments, which echoes our conclusion on the pH-dependent studies of wild-type TR. The trend of thermal stability of artificial TR pigments indicate that replacement of the β-ionone ring with phenyl or dimethylamino groups as well as linear retinal, which lacks the entire ring structure, affect the thermal stability of the protein. Therefore, it is concluded that the intact structure of the β-ionone ring of the retinal chromophore is needed for the high thermal stability of the pigment. The thermal stability of artificial TR pigments derived from synthetic retinal analogs (Fig. 5a) may indicate that alteration of the retinal conformation in the β-ionone ring region led to more efficient PSB hydrolysis, thereby reducing the protein thermal stability. Replacement of the β-ionone ring with aromatic groups as well as removing the ring presumably lessens the retinal close packing, as well as changes the protein conformation, facilitating more efficient PSB hydrolysis.

Retinal proteins experience a photocycle following light excitation^1^,^20^,^48^,^49^ which includes C_13_=C_14_ double bond isomerization of the retinal chromophore (Fig. 5d). The effects of illumination and retinal double bond isomerization on the thermal stability of the retinal proteins are yet not fully understood. To study the role played by the double bond isomerization on the thermal stability of TR, we prepared *trans*-locked TR pigment (TR-TL) by incubating *trans*-locked retinal (**6**, Fig. 5a) with TR apo-protein. Unlike native all-*trans* retinal the light induced *trans* to *cis* isomerization is prevented in this retinal. The pigment absorption band of TR-TL is red-shifted with *λ*_max_ at 550 nm, compared to the wild-type pigment (530 nm). We have recorded the absorption spectra of TR-TL at pH 6.7 and 80 °C in dark and under irradiation. The change in optical density at 550 nm reveals that the denaturation of TR-TL was accelerated following light absorption (Fig. 5e). Significant photo-catalysis of TR-TL pigment indicates that the light-induced retinal photocycle is not a prerequisite for light-induced protein conformation change. Light-induced protein conformation alterations in retinal proteins are initiated by photoisomerization of opsin-bound retinal chromophore.^1^ However, it was proposed that protein conformation alteration is triggered by light-induced charge redistribution in the retinal chromophore following photoexcitation.^50^–^53^ Polarization of the protein due to charge redistribution in the retinal chromophore was supported as well by theoretical calculations.^54^ The protein conformation change in *trans*-locked pigment of BR, in which the isomerization the C_13_=C_14_ crucial double bond is prevented, was studied and supported using atomic force sensing and electron spin resonance techniques.^52^,^53^ The possibility of isomerization of any other bond was excluded using flash photolysis and time-resolved Raman measurements.^55^,^56^ Acceleration of hydroxylamine reaction of *trans*-locked variants of halorhodopsin by irradiation supported as well that protein conformation alteration triggered by photoexcitation can catalyze the non-photochemical reaction.^50^ The above proposal was supported by recent time-resolved X-ray studies^20^ of BR photoisomerization, indicating that the protein experienced conformational alterations during the lifetime of the retinal excited state. Therefore, we may propose that the light-catalyzed thermal denaturation of TR-TL pigment is due to acceleration of the hydrolysis reaction triggered by light induced protein conformation alteration induced by the retinal excited state. The mechanism for photo-catalysis of thermal denaturation could also be explained using the protein structural alterations induced by charge redistribution in the retinal excited state.^57^–^60^ Theoretical studies^58^ on the excited state intramolecular charge transfer (ICT) in visual rhodopsin reveal that the electronic charge was translocated from its original position at the =C_13_–C_14_–NH– moiety to the –C_8_=C_9_– position in the first excited state. The excited state charge transfer process of the retinal is a fast process, occurring in few hundreds of femtoseconds in native BR, which polarizes the protein and initiates a cascade of processes, ultimately changing the protein conformation. Analysis of the kinetic of the hydroxylamine reaction in BR excluded the possibility of excited state chemical reaction.^51^ Therefore, it seems unlikely that the hydrolysis reaction takes place in the excited state. We propose that the protein alterations have much longer life-time than the excited state and allow the hydrolysis chemical reaction to take place on a lifetime longer than microseconds in the protein ground state. Initiation of protein conformational alterations in the excited state of BR was supported by a recent time-resolved X-ray study^20^ on BR photoisomerization. In the resting BR state the PSB forms hydrogen bond with a functional water molecule (Wat402) that also forms H-bonding networks with aspartic acid residues (Asp85 and Asp212). It was found that the Wat402 and PSB responds to photon absorption even before initiation of the retinal all-*trans* to 13-*cis* isomerization process. These two groups moves apart up to 0.6 Å due to light absorption. The separation is further increased to 3.25 Å at longer timescale when the retinal is isomerized. A collective motion of a group of polar residues, connected through H-bonds (Wat402, Asp212, Tyr57) were observed due to ultrafast redistribution of the positive charge towards the β-ionone ring from the PSB. Although the retinal isomerization is generally triggered by photon absorption, a novel phenomenon called the ‘thermal isomerization’ has been proposed to account for the thermal noise of visual rhodopsin.^61^–^63^ As our results show that the photo-catalysis could be achieved even in case of *trans*-locked variant of TR and previous studies excluded the possibility of isomerization of any other double bond of this retinal, we have not considered this possibility here. Therefore, we propose that protein structural alteration triggered by retinal excited state formation following light absorption is responsible for the photo-catalysis of the thermal denaturation of TR. Hay and Scrutton proposed an alternative explanation for the assisted-protein catalysis that is based on formation of light induced protein vibrations alterations.^64^ It was proposed that the fast formation of the protein vibrations observed in the picosecond and femtosecond timescale affect a chemical reaction in the protein once the vibration matches the chemical reaction energy profile, thereby reducing the energy barrier along the reaction coordinate. Although this possibility cannot be completely excluded, it seems less likely since elimination of the retinal light-induced dipole abolished the light-induced catalysis of the chemical reactions.^65^ In addition, direct light-induced protein conformation alterations were detected by AFM studies in artificial “locked” BR pigment in which double bond isomerization was prevented.^52^ Therefore, we can propose that the photo-catalysis of thermal denaturation of artificial TR pigment derived from non-isomerizable *trans*-locked retinal is achieved due to retinal excited state initiated protein structural alteration.

## Conclusion

With the aim to elucidate common factors responsible for the thermal stability of microbial retinal proteins, we have studied the effect of pH and illumination on the thermal stability of TR as well as on other retinal proteins, GR, PR and BR. The thermal denaturation of the studied retinal proteins was accelerated following light absorption. Similarity between the thermal denaturation to that of hydroxyl amine reaction and the fact that the apo-proteins are less stable than the corresponding retinal-bound opsins led us to propose that the hydrolysis of the retinal chromophore is the rate-determining step during the thermal denaturation of TR in solution. Higher thermal stability of TR in films, in which PSB hydrolysis is restricted, strongly supports this proposal. Comparison of thermal stabilities of TR, GR, PR and artificial TR pigments reveal that the PSB counter-ion protonation state does not affect the thermal stability. The TR thermal stability is affected by the retinal structure, protein conformation and the protonation state of other protein residues. The present studies suggest that the molecular mechanism for thermal denaturation proposed for TR serves as a general mechanism for all the studied retinal proteins. CD spectra of the TR at the far UV region indicate that at high temperature (above 80 °C) part of the α-helical structure of the protein turns into β-sheet structure, finally leading to disordered protein structure due to denaturation. Similar analyses reveals the apo-proteins of TR and GR are less stable than the corresponding retinal-bound opsins that echoes the results obtained from the absorption and DSF experiments. Thermal photobleaching of artificial TR pigment derived from non-isomerizable *trans*-locked retinal suggests that light-induced protein conformation changes, which are not associated with double bond isomerization, catalyze the thermal denaturation of TR. The presented results are expected to be useful in designing microbial rhodopsins with a desired stability by restricting the hydrolysis of PSB.

## Conflicts of interest

The authors declare no competing financial interest.

## Supplementary Material

Supplementary information

## References

[cit1] Ernst O. P., Lodowski D. T., Elstner M., Hegemann P., Brown L. S., Kandori H. (2014). Chem. Rev..

[cit2] Slamovits C. H., Okamoto N., Burri L., James E. R., Keeling P. J. A. (2011). Nat. Commun..

[cit3] Beja O., Spudich E. N., Spudich J. L., Leclerc M., DeLong E. F. (2001). Nature.

[cit4] Niho A., Yoshizawa S., Tsukamoto T. (2017). J. Am. Chem. Soc..

[cit5] Lutz I., Sieg A., Wegener A. A., Engelhard M., Boche I., Otsuka M., Oesterhelt D., Wachtveitl J., Zinth W. (2001). Proc. Natl. Acad. Sci. U. S. A..

[cit6] Tsukamoto T., Inoue K., Kandori H., Sudo Y. (2013). J. Biol. Chem..

[cit7] Tsukamoto T., Demura M., Sudo Y Y. (2014). J. Phys. Chem. B.

[cit8] Tsukamoto T., Mizutani K., Hasegawa K., Takahashi M., Honda N., Hashimoto N. (2016). J. Biol. Chem..

[cit9] Iyer E. S. S., Misra R., Maity A., Liubashevsky O., Sudo Y., Sheves M., Ruhman S. (2016). J. Am. Chem. Soc..

[cit10] Kato H. E., Kamiya M., Sugo S. (2015). Nat. Commun..

[cit11] Govorunova E. G., Sineshchekov O. A., Janz R., Liu X., Spudich J. L. (2015). Science.

[cit12] Ranaghan M. J., Shima S., Ramos L., Poulin D. S., Whitted G., Rajasekaran S., Stuart J. A., Albert A. D., Birge R. R. (2010). J. Phys. Chem. B.

[cit13] Wagner N. L., Greco J. A., Ranaghan M. J., Birge R. R. (2013). J. R. Soc., Interface.

[cit14] Li Y. T., Tian Y., Tian H., Tu T., Guo G. Y., Wang Q., C. Quio Y., Yang Y., L. Ren T. (2018). Sensors.

[cit15] Liu Y. M., Liu J., Mehrotra D., Liu Y., Guo Y., Baldera-Aguayo P. A., Mooney V. L., Nour A. M., Yan E. C. Y. (2013). J. Biol. Chem..

[cit16] Heyes C. D., El-Sayed M. A. (2003). J. Phys. Chem. B.

[cit17] Hubbard R. (1958). J. Gen. Physiol..

[cit18] Honda N., Tsukamoto T., Sudo Y. (2017). Chem. Phys. Lett..

[cit19] Yokoyama Y., Sonoyama M., Mitaku S. (2002). J. Biochem..

[cit20] Nogly P., Weinert T., James D. (2018). Science.

[cit21] Sheves M., Friedman N., Albeck A., Ottolenghi M. (1985). Biochemistry.

[cit22] Nakanishi K., Crouch R. (1995). Isr. J. Chem..

[cit23] Jana S., Eliash T., Jung K.-H., Sheves M. (2017). J. Phys. Chem. B.

[cit24] Tahara S., Takeuchi S., Abe-Yoshizumi R., Inoue K., Ohtani H., Kandori H., Tahara T. (2018). J. Phys. Chem. B.

[cit25] Dioumaev A. K., Wang J. M., Balint Z., Varo G., Lanyi J. K. (2003). Biochemistry.

[cit26] Vogel R., Siebert F. (2002). Biochemistry.

[cit27] Heyes C. D., El-Sayed M. A. (2001). Biochemistry.

[cit28] Yan E. C. Y., Kazmi M. A., Ganim Z., Hou J.-M., Pan D., Chang B. S. W., Sakmar T. P., Mathies R. A. (2003). Proc. Natl. Acad. Sci. U. S. A..

[cit29] Mahalingam M., Martinez-Mayorga K., Brown M. F., Vogel R. (2008). Proc. Natl. Acad. Sci. U. S. A..

[cit30] Greenfield N. J. (2006). Nat. Protoc..

[cit31] Misra R., Eliash T., Sudo Y., Sheves M. (2019). J. Phys. Chem. B.

[cit32] Tastan O., Dutta A., Booth P., Klein-Seetharaman J. (2014). Biochim. Biophys. Acta.

[cit33] Schelbach J. P., Woodall N. B., Bowie J. U., Park C. (2014). J. Am. Chem. Soc..

[cit34] Curnow P., Di Bartolo N. D., Moreton K. M., Ajoje O. O., Saggese N. P., Booth P. J. (2011). Proc. Natl. Acad. Sci. U. S. A..

[cit35] Sapra K. T., Park P. S. H., Palczewski K., Muller D. J. (2008). Langmuir.

[cit36] Yu H., Siewny M. G. W., Edwards D. T., Sanders A. W., Perkins T. T. (2017). Science.

[cit37] Holzwarth G., Doty P. (1965). J. Am. Chem. Soc..

[cit38] Sreerama N., Woody R. W. (2004). Methods Enzymol..

[cit39] London E., Khorana H. G. (1982). J. Biol. Chem..

[cit40] http://cbdm-01.zdv.uni-mainz.de/∼andrade/k2d3/, (accessed on 16.05.2019).

[cit41] Louis-Jeune C., Andrade-Navarro M. A., Perez-Iratxeta C. (2012). Proteins: Struct., Funct., Genet..

[cit42] Iwamoto M., Sudo Y., Shimono K., Kamo N. (2001). Biochim. Biophys. Acta, Biomembr..

[cit43] Tsukamoto T., Mizutani K., Hasegawa K., Takahashi M., Honda N., Hashimoto N. (2016). J. Biol. Chem..

[cit44] Shen Y., Safinya C. R., Liang K. S., Ruppert A. F., Rothschild K. J. (1993). Nature.

[cit45] Van der Steen R., Biesheuvel P. L., Mathies R. A., Lugtenburg J. (1986). J. Am. Chem. Soc..

[cit46] Wada A., Akai A., Goshima T., Takahashi T., Ito M. (1998). Bioorg. Med. Chem. Lett..

[cit47] Ahuja S., Eilers M., Hirshfeld A., Yan E. C. Y., Ziliox M., Sakmar T. P., Sheves M., Smith S. O. (2009). J. Am. Chem. Soc..

[cit48] Gozem S., Luk H. L., Shapiro I., Olivucci M. (2017). Chem. Rev..

[cit49] Kiefer H. V., Gruber E., Langeland J., Kusochek P. A., Bochenkova A. V., Andersen L. H. (2019). Nat. Commun..

[cit50] Dutta S., Hirshfeld A., Sheves M. (2015). FEBS Lett..

[cit51] Rousso I., Gat Y., Lewis A., Sheves M., Ottolenghi M. (1998). Biophys. J..

[cit52] Rousso I., Khachatryan E., Gat Y., Brodsky I., Ottolenghi M., Sheves M., Lewis A. (1997). Proc. Natl. Acad. Sci. U. S. A..

[cit53] Aharoni A., Weiner L., Ottolenghi M., Sheves M. (2000). J. Biol. Chem..

[cit54] Xu D., Martin C., Schulten K. (1996). Biophys. J..

[cit55] Ujj L., Zhou Y., Sheves M., Ottolenghi M., Ruhman S., Atkinson G. H. (1999). J. Am. Chem. Soc..

[cit56] Delaney J. K., Brack T. L., Atkinson G. H., Ottolenghi M., Steinberg G., Sheves M. (1995). Proc. Natl. Acad. Sci. U. S. A..

[cit57] Schenkl S., van Mourik F., van der Zwan G., Haacke S., Chergui M. (2005). Science.

[cit58] Andruniow T., Ferre N., Olivucci M. (2004). Proc. Natl. Acad. Sci. U. S. A..

[cit59] Groma G. I., Hebling J., Kozma I. Z., Varo G., Hauer J., Kuhl J., Riedle E. (2008). Proc. Natl. Acad. Sci. U. S. A..

[cit60] Sakai K., Vacek G., Luthi H. P., Nagashima U. (1997). Photochem. Photobiol..

[cit61] Yau K. W., Matthews G., Baylor D. A. (1979). Nature.

[cit62] Gozem S., Shapiro I., Ferre N., Olivucci M. (2012). Science.

[cit63] Yanagawa M., Kojima K., Yamashita T., Imamoto Y., Matsuyama T., Nakanishi K., Yamano Y., Wada A., Sako Y., Shichida Y. (2015). Sci. Rep..

[cit64] Hay S., Scrutton N. S. (2012). Nat. Chem..

[cit65] Zadok U., Khatchatourianst A., Lewis A., Ottolenghi M., Sheves M. (2002). J. Am. Chem. Soc..

