# Supplementary material for: Molecular mechanism for thermal denaturation of thermophilic rhodopsin
Source: Chem Sci. 2019 Jun 20;10(31):7365–74. doi: 10.1039/c9sc00855a (PMC6713869; doi:10.1039/c9sc00855a)
Supplement: Supplementary file 1 [file SC-010-C9SC00855A-s001.pdf]

Electronic Supplementary Material (ESI) for Chemical Science.
This journal is © The Royal Society of Chemistry 2019

Electronic Supplementary information

Molecular Mechanism for Thermal Denaturation of Thermophilic Rhodopsin

Ramprasad Misra[1], Amiram Hirshfeld[1], Mordechai Sheves[1*]

[1]*Department of Organic Chemistry, Weizmann Institute of Science,*

Rehovot 76100, Israel

E-mail: mudi.sheves@weizmann.ac.il

Expression and purification of TR and other microbial rhodopsins

Codon optimized TR was expressed in Luria−Bertani (LB) medium in *Esherichiacoli* (E. Coli) BL21(DE3) cells. E. Coli transformants were grown to about A_{600} = 1.30 in the presence of ampicilline (50 µg/mL) at 37 °C. The growing cells were then induced with 0.1% L(+)-arabinose and 10 µM *all-trans*-retinal for about four hours. Pink-colored cells were harvested by centrifugation at 4 °C and then resuspension with buffer S (pH 6; 50 mM MeS, 300 mM NaCl, 5 mM imidazole, 5 mM $MgCl_2$) also containing 1% n-dodecyl-β-Dmaltoside (DDM), and lysed with lysozime (0.1 mg/mL) in presence of DNAse and protease inhibitor. The resulting mixture was stirred overnight at room temperature. The extracted protein was collected as supernatant of the stirred solution after centrifugation at 18000 rpm and 4 °C for about 25 minutes. The purification of the protein was done using a Ni^{2+}-NTA histidine-tagged agarose column. First, the histidine (His)-tagged protein was washed with buffer W (pH 6; 50 mM MeS, 300 mM NaCl, 50 mM imidazol, 0.06% DDM;) and eluted with buffer E (pH 7.50; 0.06% DDM, 50 mM Tris-HCl, 300 mM NaCl, 50 mM HCl, 150 mM imidazole). The eluted protein was washed for removal of buffer E, followed by concentrating with 0.02% DDM solution that also contains 300 mM NaCl using Amicon Ultra centrifugal filter devices (300 rpm, 20 °C). Similarly, GR and PR were expressed in E. Coli and purified using similar protocol as TR [S1]. The eluted GR and PR were washed and concentrated using 0.02% DDM solution. BR was prepared using protocol reported earlier [S2]. All the proteins are stored at 4 °C before use.

Table S1: The melting temperatures (T_m, in degree Celsius) of TR at different pH, obtained through first derivatives of tryptophan fluorescence ratio at 330 and 350 nm in the 20-95 ^oC range. The T_m values of TR apo-protein at pH 6.7 are reported as well.

System/ pH	Melting temperature 1 (T_{m1}, ^oC)	Melting temperature 2 (T_{m2}, ^oC)
TR/ 3	-	81
TR/ 5	63	84
TR/ 6.7	71	86
TR/ 9	70	79
TR apo-protein/ 6.7	46	65

Table S2: Contribution of α-helix and β-sheet structures on the CD spectra in the far UV region (UV-CD) of TR, GR and their corresponding apo-proteins at different temperatures at pH 6.7. Web version of K2D3 software is used for the analyses.

**Protein**	**25 ºC**	**40 ºC**	**55 ºC**	**70 ºC**	**80 ºC**	**90 ºC**
TR	α 75.8<br>β 1.4	α 74.8<br>β 1.4	α 72.7<br>β 1.4	α 72.2<br>β 1.4	α 68.7<br>β 1.7	α 39.5<br>β 16.9
TR-apo	α 77.6<br>β 1.2	α 77.0<br>β 1.3	α 72.2<br>β 1.4	α 71.4<br>β 1.3	α 42.4<br>β 15.6	α 22.6<br>β 25.4
GR	α 81.1<br>β 1.4	α 81.1<br>β 1.2	α 81.2<br>β 1.3	α 80.6<br>β 1.3	α 38.8<br>β 18.7	-
GR-apo	α 80.8<br>β 1.3	α 80.8<br>β 1.2	α 78.0<br>β 1.3	α 56.1<br>β 9.1	α 37.1<br>β 22.3	-

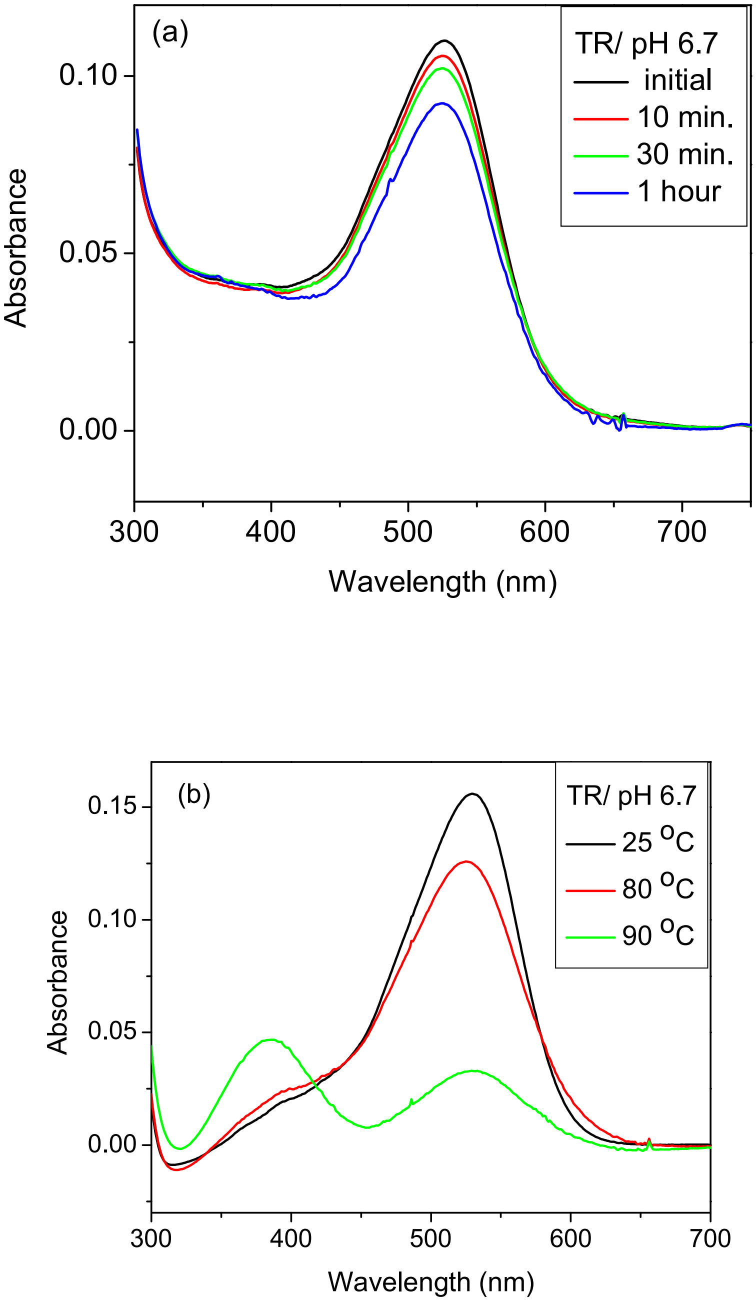

Figure S1: (a) The absorption spectra of TR at 70 ºC (pH 6.7), recorded just after reaching 70 ºC and after 10, 30 minutes and 1 hour of heating. (b) The absorption spectral change of TR due to heating to 80 and 90 ºC at pH 6.7 from 25 ºC. These spectra were recorded just after reaching the specified temperatures.

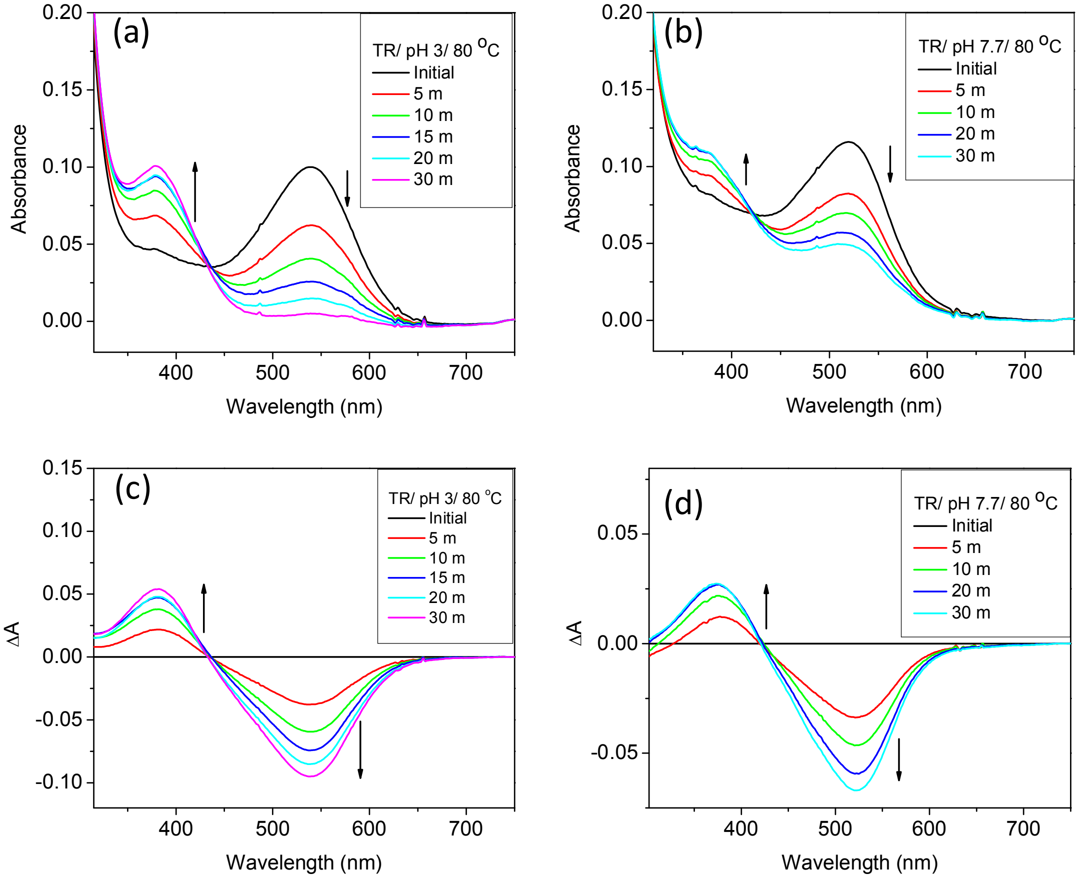

Figure S2: Change in absorption spectra of TR at different time intervals due to thermal denaturation at pH (a) 3 and (b) 7.7 at 80 ^{o}C. Panels (c-d) show the corresponding difference spectra obtained by subtracting the initial spectrum from absorption spectra recorded at different times.

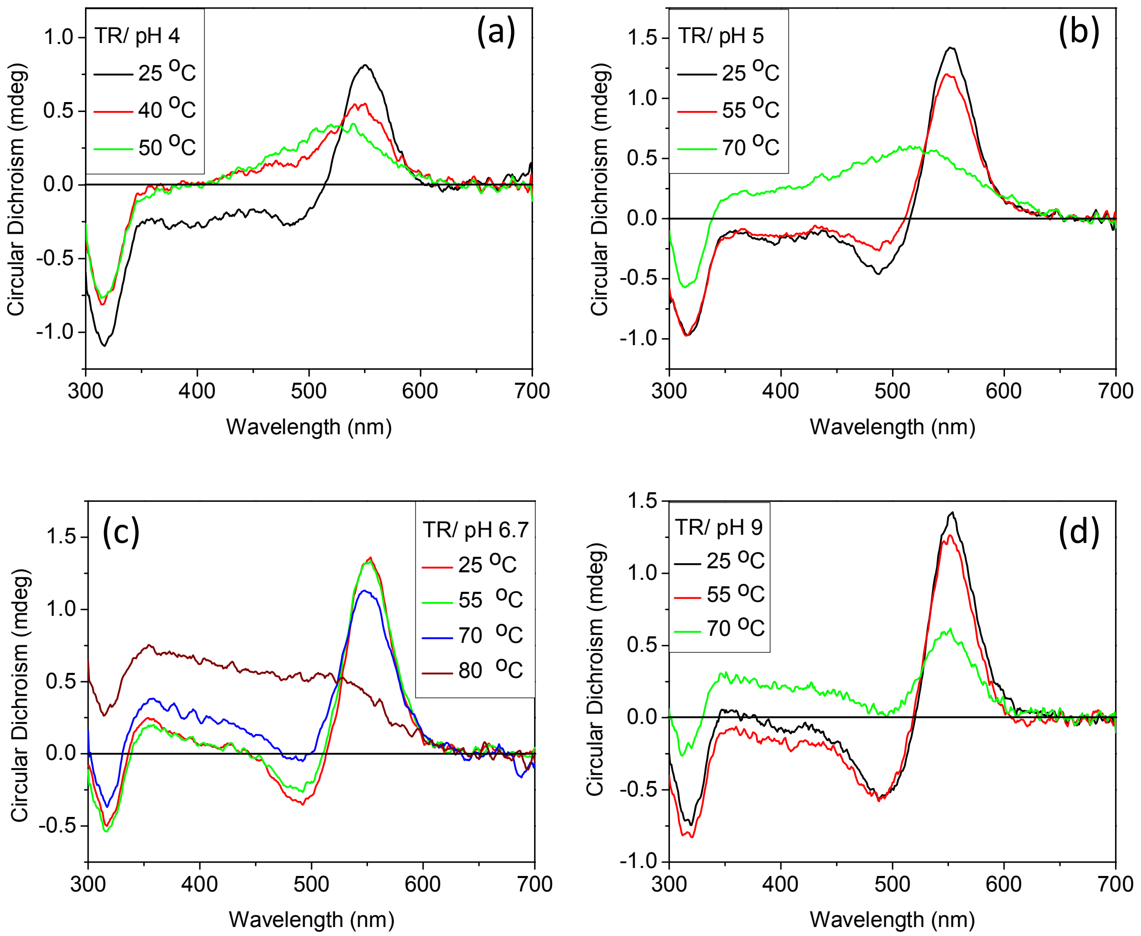

Figure S3: Temperature-dependent circular dichroism (CD) spectra of TR at pH (a) 4, (b) 5 (c) 6.7 and (c) 9. Trimeric TR shows bisignate CD spectra, possibly arising due to excitonic interaction among the retinal chromophores of the subunits. The loss of positive and negative bands of bisignate CD spectra indicate formation of the monomer.

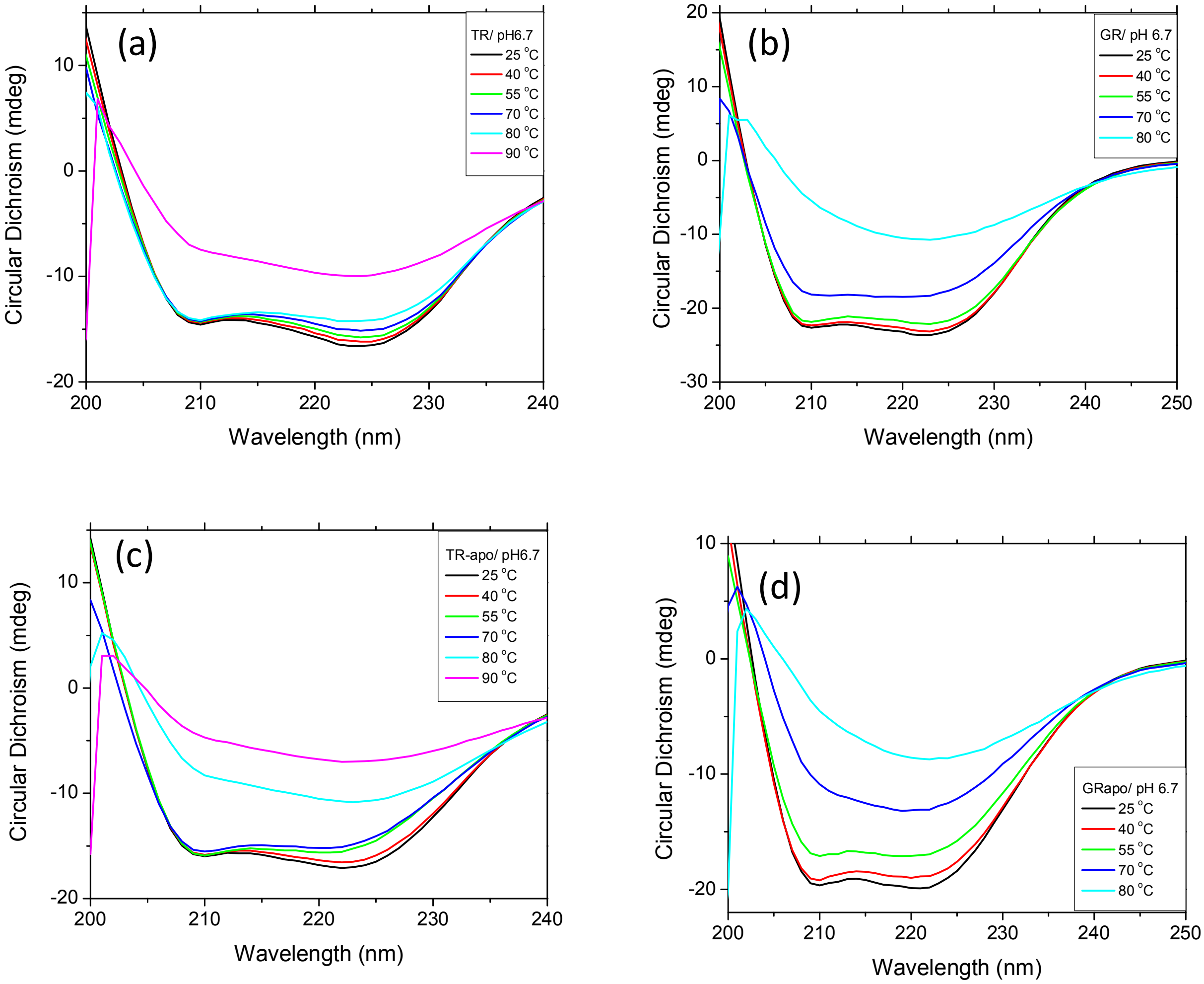

Figure S4: The effect of temperature on CD spectra of (a) TR, (b) GR and their (c-d) corresponding apo-proteins in the ultraviolet region. The apo-proteins are thermally less stable than their covalently retinal-bound pigments.

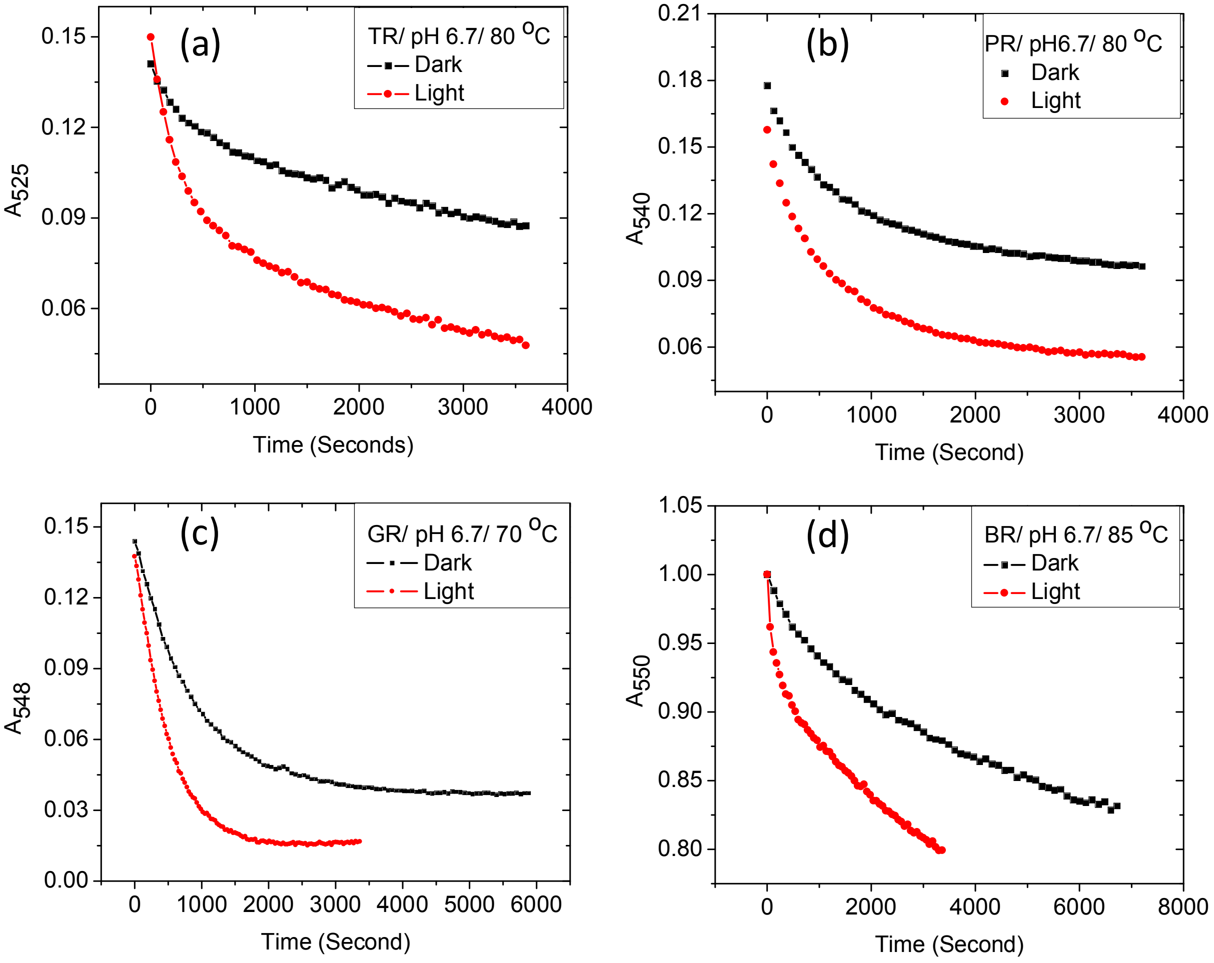

Figure S5: The effect of irradiation on the thermal stability of (a) TR and (b) PR at pH 6.7 and 80 °C. The loss of optical density of the corresponding retinal covalently-bound opsins in the dark and light are shown in black and red, respectively. Panel *c* and *d* depict the effect of irradiation on GR and BR at the same pH at 70 and 85°C, respectively.

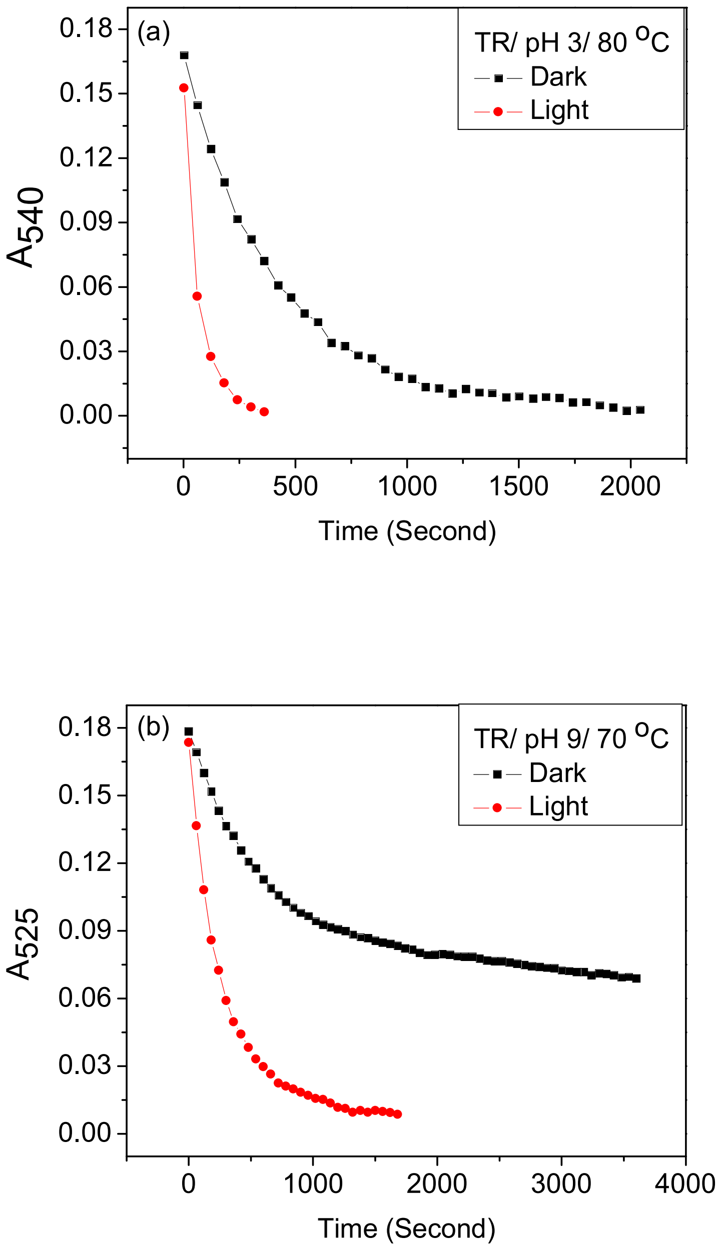

Figure S6: The effect of irradiation on the thermal stability of TR at (a) pH 3 and (b) 9 at 80 and 70ºC, respectively. The loss of absorption intensity at 540 (pH 3) and 525 (pH 9) nm with time are plotted.

References

S1. A. R. Choi, L. Shi, L. S. Brown and K. H. Jung, *PLoS One* 2014, **9,** e110643.

S2. A. Wand, B. Loevsky, N. Friedman, M. Sheves and S. Ruhman, *J. Phys. Chem. B* 2013, **117,** 4670-4679.
